# Assessing the phylogeographic history of the montane caddisfly *Thremma gallicum* using mitochondrial and restriction-site-associated DNA (RAD) markers

**DOI:** 10.1002/ece3.1366

**Published:** 2015-01-13

**Authors:** Jan-Niklas Macher, Andrey Rozenberg, Steffen U Pauls, Ralph Tollrian, Rüdiger Wagner, Florian Leese

**Affiliations:** 1 Department of Animal Ecology, Evolution and Biodiversity, Ruhr University BochumUniversitätsstraße 150, 44801, Bochum, Germany; 2 Biodiversity and Climate Research Centre (BiK-F)Senckenberganlage 25, 60325, Frankfurt am Main, Germany; 3 Senckenberg Gesellschaft für NaturforschungSenckenberganlage 25, 60325, Frankfurt am Main, Germany; 4 Working Group Limnology, University of KasselHeinrich-Plett-Straße 30, 34132, Kassel, Germany; 5 Max Planck Institute for Evolutionary BiologyAugust-Thienemann-Straße 2, 24306, Plön, Germany

**Keywords:** Biogeography, freshwater ecology, next-generation sequencing, phylogeography, Pleistocene glaciations

## Abstract

Repeated Quaternary glaciations have significantly shaped the present distribution and diversity of several European species in aquatic and terrestrial habitats. To study the phylogeography of freshwater invertebrates, patterns of intraspecific variation have been examined primarily using mitochondrial DNA markers that may yield results unrepresentative of the true species history. Here, population genetic parameters were inferred for a montane aquatic caddisfly, *Thremma gallicum*, by sequencing a 658-bp fragment of the mitochondrial *CO1* gene, and 12,514 nuclear RAD loci. *T. gallicum* has a highly disjunct distribution in southern and central Europe, with known populations in the Cantabrian Mountains, Pyrenees, Massif Central, and Black Forest. Both datasets represented rangewide sampling of *T. gallicum*. For the *CO1* dataset, this included 352 specimens from 26 populations, and for the RAD dataset, 17 specimens from eight populations. We tested 20 competing phylogeographic scenarios using approximate Bayesian computation (ABC) and estimated genetic diversity patterns. Support for phylogeographic scenarios and diversity estimates differed between datasets with the RAD data favouring a southern origin of extant populations and indicating the Cantabrian Mountains and Massif Central populations to represent highly diverse populations as compared with the Pyrenees and Black Forest populations. The *CO1* data supported a vicariance scenario (north–south) and yielded inconsistent diversity estimates. Permutation tests suggest that a few hundred polymorphic RAD SNPs are necessary for reliable parameter estimates. Our results highlight the potential of RAD and ABC-based hypothesis testing to complement phylogeographic studies on non-model species.

## Introduction

Genetic methods are widely applied to test phylogeographic hypotheses on Quaternary population dynamics, glacial/interglacial refugia, and postglacial recolonization (Hewitt 2000; Bunje 2005; Pauls et al. 2006). Traditionally, mitochondrial DNA (mtDNA) has been used as the primary genetic marker for such purposes in animals (Avise 2000). Mitochondrial genes have some obvious advantages, such as a high mutation rate, lack of recombination, and high intracellular copy number (Wilson et al. 1985). Furthermore, the common use of the *cytochrome c oxidase subunit 1* (*CO1*) gene for animal DNA barcoding has generated massive datasets as well as the necessary laboratory protocols and primers (Hebert et al. 2004). Together, these have greatly enhanced comparative phylogeographic analyses (e.g., Lehrian et al. 2009; Craft et al. 2010; Bálint et al. 2011). Therefore, until today, most studies addressing the phylogeographic history of species have been based on mtDNA (e.g., Taberlet and Bouvet 1994; Frohlich et al. 1999; Emerson et al. 2000), sometimes coupled with a few nuclear markers (e.g., Burton et al. 1994; Fijarczyk et al. 2011; Theissinger et al. 2013). Despite their advantages, mitochondrial genes have a unique evolutionary history and are therefore affected by special factors (Ballard and Whitlock 2004) that can lead to erroneous inferences (Mardulyn et al. 2011; Elbrecht et al. 2014) that thus limit their application. A reiterated plea in evolutionary biology and phylogeography is to sample as many independent genomic markers as possible to maximize the robustness and accuracy of results at the genome-wide scale (e.g., Godinho et al. 2008; Brito and Edwards 2009; Carstens et al. 2012). With the emergence of novel high-throughput sequencing methods, it is now possible to study genetic variation across whole genomes, assessing population genetic parameters from a greater number of independent nuclear loci between individuals and populations (Carstens et al. 2012; McCormack et al. 2013). One promising technique is restriction-site-associated DNA sequencing (RAD) (Baird et al. 2008; Hohenlohe et al. 2010; Peterson et al. 2012). In contrast to traditional single-gene approaches, RAD provides a low-cost, efficient method for characterization of single nucleotide polymorphisms (SNPs) at hundreds to thousands of loci across a genome. So far, RAD has been used primarily in model organisms (Hohenlohe et al. 2010, 2011, 2012; Catchen et al. 2013; Jones et al. 2013) with the advantage that generated sequences can be mapped easily against a known reference genome. However, great potential lies in the application of RAD to the population genomics of non-model species without a priori genomic information (Hohenlohe et al. 2011; White et al. 2013). A disadvantage of RAD is that no prior information on the location of loci is available without a reference genome (Cutter 2013). Furthermore, as with any marker relying on restriction endonucleases (RE), RAD markers can be affected by null alleles as a result of mutations in RE recognition sites (Arnold et al. 2013). First studies, however, have demonstrated impressive resolution when inferring the phylogeographic history and genomic parameters of both model and non-model species (Emerson et al. 2010; Hohenlohe et al. 2012; Pujolar et al. 2013; Reitzel et al. 2013; Senn et al. 2013). In a pioneering phylogeographic study with genome-scale data, Emerson et al. (2010) showed that resolution of pooled RAD data was higher than that generated from *CO1* gene data for the North American pitcher plant mosquito *Wyeomyia smithii*. However, it should be noted that by pooling samples for high-throughput sequencing in this way, no information on genetic diversity among individuals could be obtained. Analysis of single individual genotypes and heterozygosity is crucial for many basic population genetic test statistics; therefore, techniques that allow the assignment of sequences to individuals are essential for population genomic and phylogeographic studies.

**Figure 1:**
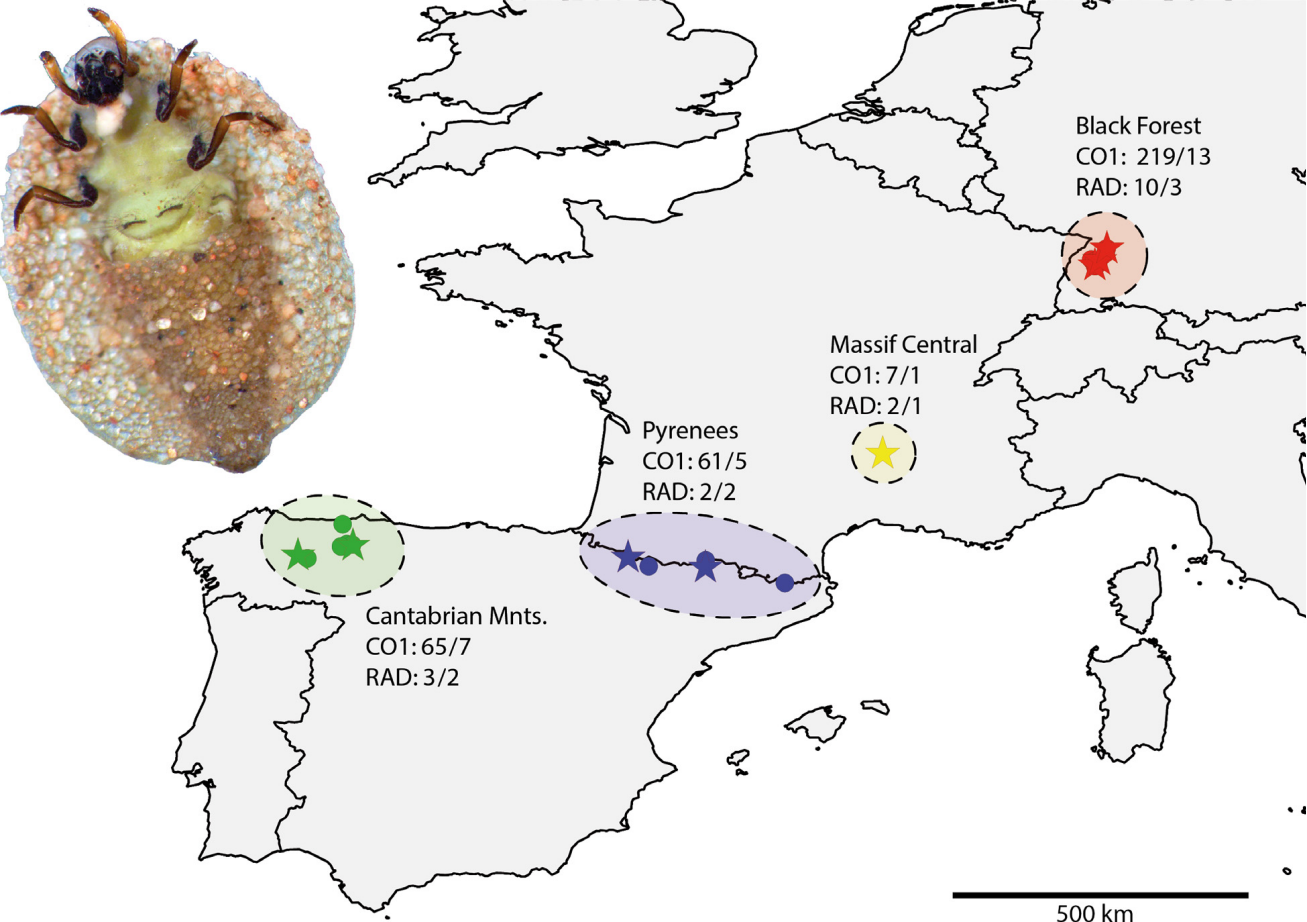
Known distribution and sampling sites of *Thremma gallicum* in this study. Stars indicate those populations from which individuals were also sampled for RAD analysis. Numbers of analyzed specimens are indicated before slash. Numbers after the slash indicate the number of sampling sites from which the sampled organisms were obtained. The picture in the upper left corner shows the ventral view of a *T. gallicum* larva.

Here, we analyzed the population structure and phylogeographic history of a European montane caddisfly species, *Thremma gallicum* McLachlan, 1880, across its distribution range, using both *CO1* and RAD data. *T. gallicum* has a highly disjunct distribution range (Fig.1) (Kehl 2005). The species inhabits perennial headwater streams in the Cantabrian Mountains (Spain), Pyrenees (Spain & France), Massif Central (France), and a small area of the northern Black Forest (Germany). For unknown reasons, it is absent from some areas where suitable habitat is available, for example, the eastern Cantabrian Mountains and the Vosges in France. Some, but not all, of the potential areas and habitats that do not harbor *T. gallicum* populations were extensively glaciated during the Pleistocene (Buoncristiani and Campy 2004; Ehlers and Gibbard 2004; Hughes et al. 2006; Hughes and Woodward 2008; Serrano et al. 2012; Jiménez-Sánchez et al. 2013). It thus remains unknown whether populations are connected via gene flow at present, whether they are the result of a recolonization from south to north after the last glacial maximum, or whether they persisted during glacial periods in independent refugia. If northern populations were recently recolonized from ancestral southern populations, one would expect a latitudinal gradient in genetic diversity (e.g., southern richness and northern purity, see Hewitt 2000; Lacourse et al. 2005; Soltis et al. 2006; Fulton et al. 2013; Salvi et al. 2013 for examples). If, however, ancestral refugial populations and extant populations persisted independently over glacial cycles, one would predict that differentiation between regions would lead to high regional endemism. For cold-adapted freshwater species, survival in periglacial northern refugia was postulated by Malicky (1983) and recent studies using mtDNA (e.g., Pauls et al. 2006; Lehrian et al. 2010) or a combination of mtDNA and microsatellites (Theissinger et al. 2013) have provided strong evidence for this *in situ* survival hypothesis for freshwater taxa. In this study, we applied *CO1* as well as individualized RAD-based SNP genotyping to test competing hypotheses on the origin and dispersal of *T. gallicum* populations across its current distribution range using approximate Bayesian computation (ABC) simulations. In particular, we addressed the question of whether or not RAD data from many loci across the genome for few individuals can significantly improve the resolution of phylogeographic studies based on single locus *CO1* data from many individuals. Furthermore, we estimated the number of RAD loci necessary to obtain robust estimates of population genetic parameters.

## Materials and Methods

### DNA extraction, PCR, and *CO1* sequencing

Genomic DNA (gDNA) was extracted from 352 *T. gallicum* specimens (Table1) using the Qiagen DNeasy Kit (Qiagen, Hilden, Germany) according to the manufacturer's recommendations. A 658-bp barcoding fragment of *CO1* was amplified for all 352 specimens and one specimen of *Thremma tellae* for use as an outgroup. Primers used were LCO1490 and HCO2198 (Folmer et al. 1994). Individual 25-*μ*L reactions contained 1 × HotMaster Buffer (5Prime, Hamburg, Germany), 0.2 mmol/L dNTPs, 0.5 *μ*mol/L of each primer, 0.025 U/*μ*L HotMaster Taq (5Prime), 1–3 *μ*L DNA (5–20 ng), and a sufficient volume of double-distilled H_2_O to yield a final volume of 25 *μ*L. Bidirectional sequencing was performed by GATC-Biotech (Konstanz, Germany) and Macrogen (Seoul, Korea).

**Table 1:** Sampling sites and specimens per region for the *CO1* and RAD datasets (for details, see Table S1)

Mountain range	Sampling sites (*CO1*)	Specimens (*CO1*)	Sampling sites (RAD)	Specimens (RAD)
Black Forest	13	219	3	10
Massif Central	1	7	1	2
Pyrenees	5	61	2	2
Cantabrian Mountains	7	65	2	3

### *CO1* analyses

Forward and reverse reads were assembled using Geneious Pro 5.6.5 (Drummond et al. 2011) and manually checked for erroneous base calls. A multiple sequence alignment was generated using MAFFT v. 6.814b (Katoh et al. 2002) as implemented in Geneious. A *CO1* parsimony network was computed using TCS v. 1.2.1 (Clement et al. 2000) based on the alignment of 352 sequenced specimens. The connection limit was set to ≥95%. Analysis of molecular variance (AMOVA) was performed with Arlequin v. 3.5 (Excoffier and Lischer 2010) to assess genetic differentiation and partitioning of genetic variation (see Figure S1). Arlequin was also used for the computation of haplotype and nucleotide diversity.

### Creation and sequencing of RAD libraries

**Figure 2:**
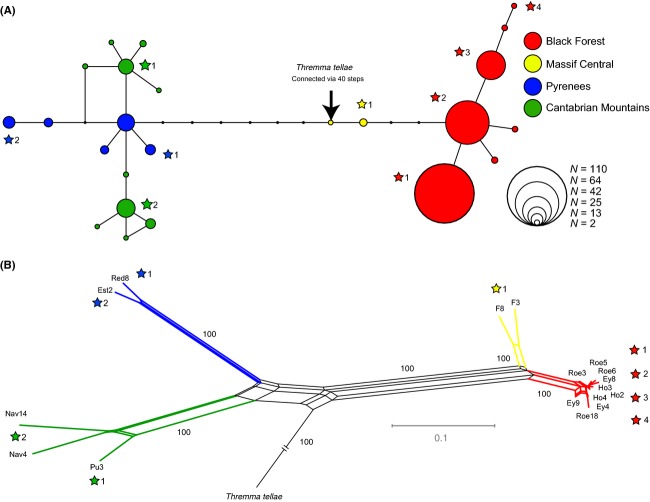
(A) Statistical parsimony network for the 658-bp *CO1* fragment analysis based upon 352 specimens. Circle diameter is proportional to number of specimens with that haplotype. The number of black dots between haplotypes corresponds to extinct or unsampled haplotypes. (B) Neighbor-net based upon 12,514 loci (15,305 SNPs) obtained through RAD sequencing of *Thremma gallicum* specimens and one outgroup specimen of *Thremma tellae*. Bootstrap values above 85 are shown on branches. Stars indicate to which *CO1* haplotype the specimens sequenced with RAD belong.

RAD libraries were prepared for 2–10 individuals per geographic region (Table1), representing all of the haplotype groups found among specimens in the *CO1* dataset (Fig.2). Libraries were prepared following the double-digest RAD (ddRAD) protocol described by Peterson et al. (2012). Briefly, gDNA was extracted from a subset of the individuals examined in the *CO1* analysis and treated with RNase (1 *μ*L of 10 *μ*g/mL RNAse per 80 *μ*L of sample). Next, 200 ng of purified gDNA was digested with 15 U (1.5 *μ*L) of the frequent cutter *Csp6I* (recognition site G↑TAC) and 15 U (1.5 *μ*L) of the rare cutter *NsiI* (recognition site ATGCA↑T; both enzymes: Thermo Fisher Scientific, Schwerte, Germany) in 3 *μ*L FastDigest buffer. The volume was filled to 30 *μ*L with H_2_O. Digestion success was checked on 1.5% TBE agarose gel using 3 *μ*L of the digested sample. The remaining 27 *μ*L was purified with the Qiagen Reaction Cleanup Kit according to the manufacturer's protocol. After this step, the universal P2 adapters (5′-AGATCGGAAGAGCGGTTCAGCAGGAATGCCGAG-3′) (1.7 *μ*L, 10 *μ*mol/L;annealing to *Csp6I*-cut ends) and customized P1 adapters (5′-ACACTCTTTCCCTACACGACGCTCTTCCGATCTxxxxxxTGCA-3′) (0.6 *μ*L, 1 *μ*mol/L; annealing to *NsiI*-cut ends), which contained individual 6-bp barcode sequences (Table S2), were ligated to the fragments. Ligation was performed in 1 × T4 ligase buffer and 0.5 *μ*L of high-fidelity T4 ligase (2,000,000 U/mL) (New England Biolabs, Frankfurt a. M., Germany) at 16°C for 2 h. The complete ligation reaction was run on a 1.5% TAE agarose gel, stained with 1% SYBR Green dye (Sigma-Aldrich, St. Louis, MO, USA) for 10 min, and visualized on a blue light illuminator. Gel regions in the 250 to 500-bp range were excised and purified using the Qiagen Gel Purification Kit. PCR was run with 25 *μ*L of sample, 10 *μ*L Q5 buffer (New England Biolabs), 5 *μ*L P5 primer (AATGATACGGCGACCACCGA, 10 *μ*mol/L), and 5 *μ*L P2_rev primer (CAAGCAGAAGACGGCATACGA, 10 *μ*mol/L), 0.5 *μ*L Q5 Taq 2U/uL (New England Biolabs), and 5 *μ*L dNTPs (2 mmol/L). PCR started with an initial denaturation step at 98°C for 30 sec followed by 16 cycles of denaturation at 98°C for 10 sec, annealing at 65°C for 30 sec, and elongation at 72°C for 30 sec, followed by a final elongation step of 72°C for 5 min. After PCR, the samples were purified with the Qiagen Reaction Cleanup Kit and eluted in 30 *μ*L H_2_O. Seventeen individual sample concentrations were quantified and approximately 25 ng of DNA per sample was pooled for sequencing by GenXPro (Frankfurt/Main, Germany) on an Illumina HiSeq 2000.

### Processing of sequenced RAD tags

Sequenced RAD tags were processed with Stacks v. 0.99992 (Catchen et al. 2011, 2013). Quality filtering was performed with the process_radtags script with default settings. As no reference genome for *Thremma gallicum* exists, we performed a *de novo* assembly of the reads (module: ustacks). Information on the modules of the Stacks pipeline can be found in Catchen et al. (2011, 2013). We extended the graphical user interface of Stacks to initiate data-cleaning steps and direct assembly on our local server via web forms, as well as to control the process and management of input and output files. We tested three different combinations of settings for filtering the reads and assessed the effects as follows. The minimum stack depth (the -m option for ustacks) was set to 3, 5, and 10; the maximum intra-individual distance (-M, ustacks) to 2, 3, and 4; and the number of mismatches tolerated for the alignment of secondary reads (-N, ustacks) to 2, 3, and 4, respectively. This last range of -N was the same for the number of mismatches allowed when building the catalog (-n, cstacks). The deleveraging (-d) algorithm was used as per default. Using a Perl script (stacks2fasta.pl, see, https://github.com/evoeco/radtools), we created an output file that was loaded into Geneious for further processing and export into other formats. The Stacks parameters chosen were as follows: minimum stack depth (-m), 3; maximum intra-individual distance (-M), 2; maximum number of mismatches for the alignment of secondary reads (-N), 2; and number of mismatches when building the catalog (-n), 2. All loci with more than two alleles were removed, as they may represent paralogous genes. For the final dataset, only loci sequenced with ≥5× coverage and ≤2 SNPs that were present in at least nine analyzed specimens (i.e., 50% of all specimens) were considered, resulting in 12,514 loci containing 15,305 SNPs. For phylogeographic hypothesis testing with the software DIYABC 2.03 (Cornuet et al. 2014, we included only loci for which at least eleven specimens had data (resulting in a 451 loci/525 SNP dataset).

### SNP analyses

The number of loci and SNPs, homo- and heterozygosity within individuals, and differentiation between populations were calculated using the filtered RAD dataset with 12,514 loci. A neighbor net calculated from these data was constructed with Splitstree v. 4.13.1 (Huson and Bryant 2006). Pairwise F_ST_ values were estimated using Genepop 4.2.2 (Rousset 2008). To assess the level of heterozygosity within individuals and populations, we used the stacks2fasta script. To test the number of loci needed to obtain reliable estimates of heterozygosity, we performed different bootstrap permutation tests randomly drawing from 10 to 1000 loci from the total RAD dataset in locus increments of 10 with another custom script (arpsampler.pl, available also from https://github.com/evoeco/radtools). From the resulting datasets, we calculated the average heterozygosity and standard deviation for the four geographically defined populations (1000 total permutations, 10-locus increments) and two representatives of each population with the highest and lowest heterozygosity (100 permutations to limit computational time). Graphs were visualized using the SVGGraph library 2.13 (Goat1000 2013).

To assess the number of loci required to obtain the correct population topology, we compared phylogenetic trees inferred from permuted alignments (concatenated SNPs) to a reference phylogeny computed for the full (12,514 loci) dataset. Permutations were designed in a similar manner to that described above with the exception that the range of alignment sizes was in 100-loci increments and alignment length was extended to 5000 loci. To facilitate comparison of tree distances, the complexity of the reference tree was reduced to an unrooted, partially resolved tree, consisting of clades reflecting individual populations and the main subdivision of the two southern and the two northern populations. Specimens from the Massif Central were not united in a single clade, as placement of this grouping on the dichotomous trees was not consistent. The constrained topology of the reference tree was thus designed as follows: (((Black Forest), Massif Central 1, Massif Central 2), (Pyrenees, Cantabrian Mountains)). For each alignment, a neighbor-joining tree based on Kimura-2-parameter distances was reconstructed and the symmetric-difference metric (Penny and Hendy 1985) was calculated as a measure of the difference between each of these trees and the reference topology. These calculations were performed in PAUP v. 4.0b10 (Swofford 2002) using a custom Perl script. The resulting distances were rescaled to the range [0; 1] based on the theoretical minimum and maximum of the metric for a given reference tree, and the mean values were plotted against alignment sizes. We estimated the convergence point as the number of loci required for tree calculation to reduce the average distance of the calculated trees to the reference tree to below 0.025 (5% of the initially expected random rescaled symmetric distance of 0.5).

### Population history

The software package BEAST (v. 1.8.0, Drummond et al. 2012) was used to estimate divergence times from the *CO1* dataset. We applied a strict molecular clock with a rate of 3.54% divergence per million years (after Papadopoulou et al. 2010). We ran BEAST for 30 million generations with default settings. HKY was determined to be the best substitution model using jModelTest 2 (Darriba et al. 2012). Parameter convergence and effective sample size (>200) was verified using Tracer v.1.5. (Rambaut et al. 2013) 7.5 million generations were discarded as burn-in, and a consensus tree was calculated using TreeAnnotator v. 1.8 and visualized in FigTree v. 1.4 (Rambaut 2007).

**Figure 3:**
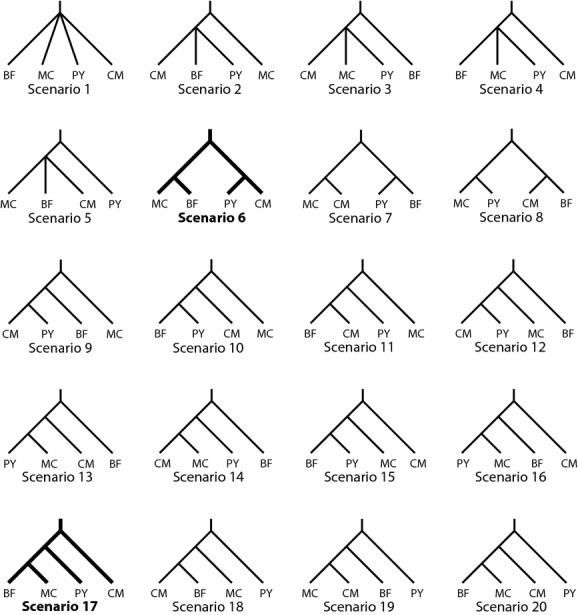
Phylogeographic hypotheses explicitly tested using approximate Bayesian computation simulations in DIYABC. Abbreviations: BF = Black Forest, MC = Massif Central, PY = Pyrenees, CM = Cantabrian Mountains. Scenario 6: Ancestral split between populations from Massif Central/Black Forest and Cantabrian Mountains/Pyrenees. Scenario 17: Gradual colonization from the Cantabrian Mountains toward the Pyrenees to the Massif Central and from there to the Black Forest.

In order to test specific hypotheses of demographic history for this species, we utilized an ABC approach in the software DIYABC v. 2.0.3 (Cornuet et al. 2014). In total, we estimated the posterior probability of 20 different phylogeographic scenarios for *T. gallicum* (Fig.3) for both the *CO1* dataset and a subset of 525 RAD SNPs that were present in at least 11 individuals. For both datasets, priors were set to follow log-uniform distributions. Priors for effective population sizes (*N*_*e*_) ranged from 1 to 400,000 for the RAD dataset and from 0.25 to 100,000 for the mitochondrial dataset (given the fourfold smaller effective number of the mitochondrial genome). Priors for the generation times between divergence events were set to 1000 to 10 million. Simulations were run for 5 million generations after which summary statistics were generated for: (1) mean of pairwise differences, (2) variance of pairwise differences, (3) Tajima's D, (4) number of segregating sites, (5) mean of pairwise differences (w), (6) mean of pairwise differences (b), and (7) *F*_ST_. For the RAD dataset, priors for the mutation rate were set to a mean of 10^−7^ (minimum 10^−8^, maximum 10^−6^). The number of invariant sites was set to 98% as we allowed for up to two SNPs per 100-bp stack. For the mitochondrial dataset, the mutation rate was left on default setting (mean of 10^−6^, maximum 10^−5^, minimum 10^−7^) and the number of invariant sites was set to 95%. All other settings were left as suggested in the DIYABC documentation for RAD and mitochondrial datasets, respectively. Posterior probabilities of scenarios were calculated by direct estimation and logistic regression considering between 500 and 50,000 datasets that were closest to the observed values.

## Results

### Population structure inferred from *CO1* dataset

In the 658-bp alignment of 352 *CO1* sequences, 28 variable positions were present. The uncorrected pairwise genetic distances between all sequenced specimens from the Black Forest, Massif Central, Pyrenees, and Cantabrian Mountains ranged between 0 and 2.6%. The *CO1* haplotype network (Fig.2A) consisted of 24 haplotypes and revealed a clear association between *CO1* haplotype relatedness and the four major regions of the *T. gallicum* distribution (see also Isolation-by-Distance analysis in Fig. S2). In fact, all haplotypes were found to be endemic to the four sampled mountain ranges, and *F*_ST_ values were high and significant between mountain ranges (Table2 and Figure S1). The seven haplotypes from the Black Forest were separated by three to seven substitutions (0.46–1.06%) from both haplotypes from the Massif Central. Haplotypes from the Massif Central were separated by 7–11 substitutions (1.06–1.67% uncorrected p-distance) from haplotypes from the Pyrenees and by 8–11 substitutions (1.22–1.67%) from those from the Cantabrian Mountains. Specimens from the Cantabrian Mountains formed two haplotype groups, separated by at least two substitutions. Both groups were more closely related to some specimens from the Pyrenees than to each other (Fig.2A). The specimens from the Pyrenees formed two groups as well: Two haplotypes were connected to both the other haplotypes from the Pyrenees and one group from the Cantabrian Mountains over a distance of two substitutions. *Thremma tellae* connected to the *T. gallicum* network via 40 steps (see Fig.2A).

**Table 2:** Genetic differentiation estimates (*F*_ST_ values) between regions for the 658-bp *CO1* fragment (below diagonal) and the RAD data (above diagonal). Significance was assessed using 1000 permutations for *CO1* and using exact G-tests and a Chi-square test averaged over loci for the RAD data

	Black Forest	Massif Central	Pyrenees	Cantabrian Mountains
Black Forest		0.37*	0.68*	0.64*
Massif Central	0.78*		0.55*	0.47*
Pyrenees	0.89*	0.83*		0.39*
Cantabrian Mountains	0.89*	0.83*	0.34*	

All results are highly significant (*P < 0.01).

### Population structure inferred from the RAD dataset

Sequencing success varied among the different libraries (Table3). In total, we found 12,514 homologous loci (15,305 SNPs) present in at least nine of the 17 specimens across all eight populations (Table1, Table S1). Relative frequencies of nucleotides in the full alignment were A (26.9%), C (22.8%), G (22.5%), T (27.8%). In the neighbor-net network (Fig.2B), specimens from the Cantabrian Mountains and Pyrenees clustered on distinct branches, indicating that these populations are clearly separated. However, populations from the Massif Central and Black Forest, although geographically far apart, clustered closer together. Bootstrap support for all branches between mountain ranges was 100. One specimen of *T. tellae* was used as the outgroup; it shared only 204 SNPs with *T. gallicum*.

**Table 3:** Posterior probability estimates (direct and logistic) for 20 phylogeographic scenarios tested in DIYABC (see Fig.3). Estimates were obtained for both the *CO1* and RAD datasets. The best models are highlighted with bold font

	RAD dataset		*CO1* dataset	
Scenario	Direct regression	Logistic regression	Direct regression	Logistic regression
1	0.1240 [0.0000, 0.4129]	0 [0.0000, 0.9310]	0.0160 [0.0000, 0.1260]	0.0001 [0.0000, 0.3486]
2	0.0080 [0.0000, 0.0861]	0 [0.0000, 0.7989]	0.0320 [0.0000, 0.1863]	0 [0.0000, 0.3485]
3	0.0100 [0.0000, 0.0972]	0 [0.0000, 0.7989]	0.0500 [0.0000, 0.2410]	0.0001 [0.0000, 0.3486]
4	0.1960 [0.0000, 0.5440]	0 [0.0000, 0.7989]	0.0000 [0.0000, 0.0000]	0 [0.0000, 0.3485]
5	0.0240 [0.0000, 0.1582]	0 [0.0000, 0.7989]	0.0040 [0.0000, 0.0593]	0 [0.0000, 0.3485]
6	0.0860 [0.0000, 0.3317]	0.0713 [0.0000, 0.8078]	**0.3900 [0.0000, 0.8175]**	**0.9902 [0.9867, 0.9936]**
7	0.0120 [0.0000, 0.1074]	0 [0.0000, 0.7989]	0.0020 [0.0000, 0.0412]	0 [0.0000, 0.3485]
8	0.0040 [0.0000, 0.0593]	0 [0.0000, 0.7989]	0.0020 [0.0000, 0.0412]	0 [0.0000, 0.3485]
9	0.0000 [0.0000, 0.0000]	0 [0.0000, 0.7989]	0.1740 [0.0000, 0.5063]	0.002 [0.0000, 0.3503]
10	0.0000 [0.0000, 0.0000]	0 [0.0000, 0.7989]	0.0060 [0.0000, 0.0737]	0 [0.0000, 0.3485]
11	0.0000 [0.0000, 0.0000]	0 [0.0000, 0.7989]	0.0040 [0.0000, 0.0593]	0 [0.0000, 0.3485]
12	0.0020 [0.0000, 0.0412]	0 [0.0000, 0.7989]	0.2980 [0.0000, 0.6989]	0.0063 [0.0000, 0.3553]
13	0.0040 [0.0000, 0.0593]	0 [0.0000, 0.7989]	0.0060 [0.0000, 0.0737]	0 [0.0000, 0.3485]
14	0.0000 [0.0000, 0.0000]	0 [0.0000, 0.7989]	0.0060 [0.0000, 0.0737]	0 [0.0000, 0.3485]
15	0.0420 [0.0000, 0.2178]	0 [0.0000, 0.7989]	0.0000 [0.0000, 0.0000]	0 [0.0000, 0.3485]
16	0.0540 [0.0000, 0.2521]	0 [0.0000, 0.7989]	0.0000 [0.0000, 0.0000]	0 [0.0000, 0.3485]
17	**0.2620 [0.0000, 0.6474]**	**0.8174 [0.6590, 0.9758]**	0.0020 [0.0000, 0.0412]	0.0009 [0.0000, 0.3493]
18	0.0160 [0.0000, 0.1260]	0 [0.0000, 0.7989]	0.0020 [0.0000, 0.0412]	0 [0.0000, 0.3485]
19	0.0020 [0.0000, 0.0412]	0 [0.0000, 0.7989]	0.0020 [0.0000, 0.0412]	0 [0.0000, 0.3485]
20	0.1540 [0.0000, 0.4704]	0.1114 [0.0000, 0.8054]	0.0040 [0.0000, 0.0593]	0.0004 [0.0000, 0.3488]

### Genetic diversity based on *CO1*

Haplotype diversity was higher in the Pyrenees (H = 0.68), Cantabrian Mountains (H = 0.63), and Black Forest (H = 0.67) than in the Massif Central (H = 0.29). Nucleotide diversity was highest in the Cantabrian Mountains (*π = *0.0031) and lowest in the Massif Central (*π = *0.0005). The Pyrenees and Black Forest populations had intermediate values (*π = *0.0028 and *π = *0.0017, respectively).

### Genetic diversity based on RAD

**Figure 4:**
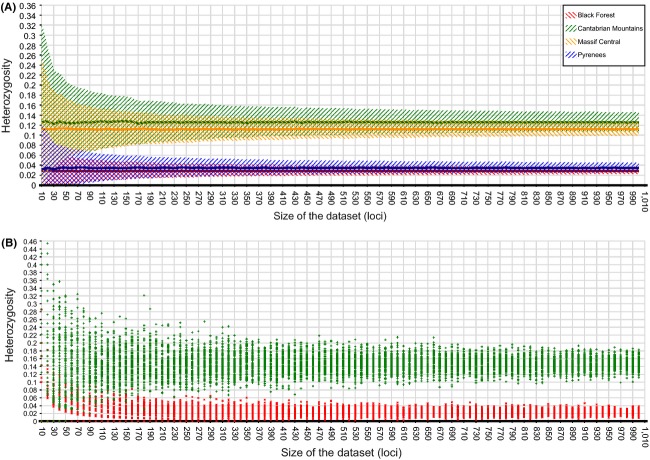
Analysis of heterozygosity averages and standard deviations (*y*-axis) with increasing number of RAD loci (10–1000, *x*-axis). (A) Averages and standard deviations of heterozygosity values for populations from each of the four mountain ranges, based on resampling the number of loci 1000 times (permutation test). (B) Visualization of 100 individual heterozygosity estimates per locus for two specimens representing the most (Cantabrian Mountains, sample Nav14) and least (Black Forest, sample Roe6) diverse populations.

Average heterozygosity was clearly lower in the Black Forest (2.6% ± 0.60) and Pyrenees (3.5 ± 1.02%) than in the Cantabrian Mountain (12.6 ± 1.84%) and Massif Central (11.4 ± 0.11%) populations. The relative patterns of heterozygosity remained similar across the parameter ranges tested. However, for the full dataset, that is, including loci found in single individuals, heterozygosity of the Cantabrian Mountains population was even higher than estimates from the current dataset, where at least nine individuals had to be present for a locus to be included. In order to assess reliability and stability of these estimates, we plotted average heterozygosity (±SD) for each population based upon permuted datasets of different sizes (10–1000 loci) (Fig.4A). While standard deviations for small datasets were large if the number of the loci sampled was below 100, such that even two populations with contrasting heterozygosity overlap, convergence was apparent for datasets greater than 250 loci.

**Figure 5:**
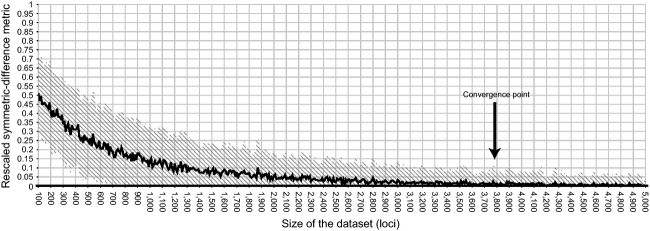
Symmetric-difference distances (*y*-axis) between trees reconstructed for permuted datasets of 100–5000 loci (*x*-axis) and the reference tree, reflecting major groupings of the populations studied. The arrow indicates the point where the average distance was constantly below a value of 0.025 (5% of the initially expected rescaled distance of 0.5).

When comparing heterozygosity estimates for two specimens representing the populations with the highest (Cantabrian Mountains, sample Nav14, heterozygosity 14.5%) and lowest (Black Forest, sample Roe6, heterozygosity 2.5%) heterozygosity, no overlap in standard deviation was found when sampling more than 290 loci (Fig.4B). Comparing normalized tree distances between reconstructed topologies (based on 100–5000 loci) and the optimal reference tree (based on the full set of 12,514 loci) revealed that 3780 loci are required to reach convergence (Fig.5).

### Population history

Molecular clock analyses of the *CO1* dataset suggest that the Iberian and French/German *T. gallicum* populations diverged approximately 0.925 million years ago (mya; 95% HPD: 0.4254–1.5288 mya). The split between the Massif Central and Black Forest populations dated to 0.2101 mya (95% HPD: 0.0724–0.3802 mya). Populations from the Cantabrian Mountains and the Pyrenees were not reciprocally monophyletic and thus divergence dating was performed for the two main splits that occurred between populations on the Iberian Peninsula (split 1: 0.209 mya [95% HPD: 0.3712–0.0747 mya]; split 2: 0.1498 [95% HPD: 0.2387–0.0475 mya], Table S3, Fig. S3).

The ABC simulations performed on the RAD data add support to the hypothesis that the Cantabrian Mountains (CM) host the oldest population (scenario 17, logistic regression: 0.8174, see Table3), which is consistent with high heterozygosity values. Derived from this are first the Pyrenees (PY) population and then the Massif Central (MC)/Black Forest (BF) population (Supporting Information 7). The second best hypothesis posits the Pyrenees populations as the most ancestral (PY,(CM,(MC,BF))), (Scenario 20, logistic regression 0.1114), and the third only resolves the two larger clusters ((CM,PY),(MC,BF)) (Scenario 6, logistic regression 0.0713).

The ABC simulations for the *CO1* data mostly support the divergence between the two southern and two northern populations as described in scenario 6 ((CM,PY),(MC,BF)) (logistic regression: 0.9902). The second and third most supported scenarios, 9 (logistic regression: 0.002) and 12 (logistic regression: 0.0063) (Table3), suggest that populations from the Massif Central (scenario 9) and Black Forest (scenario 12) are the most basal populations with the Cantabrian Mountains and Pyrenees populations being derived from these.

## Discussion

### Phylogeographic history of *Thremma gallicum*

The Iberian Peninsula was a major refuge for different terrestrial species during the Quaternary ice ages and now harbors a relatively large number of species with high genetic diversity compared with northern regions of Europe (Hewitt 2000; Gomez et al. 2007; González-Sampériz et al. 2010). Several studies, however, have shown that freshwater species also persisted in northern refugia (see Schmitt and Varga 2012), particularly in periglacial zones (Malicky 1983, 2006; Pauls et al. 2006; Theissinger et al. 2013). These dinodal species survived glacial maxima within the periglacial area by retreating to permanently flowing streams (Malicky 1983). Rising temperatures during interglacial times drove many species to extinction in low-lying areas or forced them to retreat to nearby mountain ranges where suitable habitat was still available. This theory can explain the present-day disjunct distribution ranges of such species (Hewitt 2000; Schmitt et al. 2010; Stewart et al. 2010; Theissinger et al. 2013). This pattern may also be true for *T. gallicum*: Both the *CO1* dataset (no haplotype sharing, high *F*_ST_ values between regions, significant isolation-by-distance) and the RAD dataset (high *F*_ST_ values, long branches) support a history of long-term isolation of geographically distinct populations from the four mountain ranges. In the *CO1* dataset, the divergence between populations from the Iberian Peninsula (Pyrenees and Cantabrian Mountains) was rather shallow, implying a relatively recent split between them. The paraphyletic Cantabrian Mountain specimens (Fig.2A) suggest the presence of two possible refugial areas. Conversely, although based upon only 17 specimens, the RAD data clearly support the geographic subdivision of *T. gallicum* into four distinct genetic groups that correspond to the sampled mountain ranges. This pattern supports the assumption that gene flow has been interrupted between ranges for an extended period of time. While populations from the Cantabrian Mountains and Pyrenees diverged earlier from a common ancestor (Fig.2B), the divergence of populations from the Massif Central and Black Forest is more recent. Molecular dating of such divergence events can only be performed with calibration points or reported clock rates. While no such information exists for the RAD data, clock rates for the mitochondrial *CO1* gene have been reported and using an insect-specific clock rate (Papadopoulou et al. 2010), we predict that the ancestral split between the southern and northern populations likely occurred during the late Calabrian stage of the Pleistocene. Divergence between Massif Central and Black Forest populations and from Cantabrian Mountain and Pyrenees populations dates roughly to the time of the Saale glaciation (0.30–0.13 mya). Strong shifts in vegetation and climate zones that occurred during glaciations could explain the divergence of these formerly connected populations, and this supports the hypothesis that the Black Forest was not recolonized by *T. gallicum* after the last glacial maximum from the Massif Central, but more likely served as a northern refugium as reported for other aquatic insects (e.g., *Rhyacophila aquitanica*, Bálint et al. 2008). No single split between populations from the Cantabrian Mountains and the Pyrenees could be detected for the *CO1* data, possibly hinting at several (re)colonization events of high-altitude habitats after differe nt glaciations. Molecular clock estimates are, however, error prone (e.g., Ho 2007), and thus, results must be regarded with caution. At minimum though, all splits seem to clearly predate the last glacial maximum, supporting the idea of independent glacial refugia for one or more glacial cycles.

As described earlier, reliance on a single molecular marker especially one with a myriad of challenges (reduced effective population size, sex bias, etc.) for reconstruction of demographic history is a concern. Thus, we also addressed similar hypotheses with our RAD dataset, which supports two populations with high genetic diversity (Cantabrian Mountain and Massif Central) and two with low (Pyrenees and Black Forest). Both the Black Forest and Pyrenees populations may be genetically less diverse due to recolonization from lower altitudes. Analogously, an upward recolonization in the Pyrenees was observed for the high-altitude caddisfly *Drusus discolor* (Pauls et al. 2006). However, more specimens for the Pyrenees population are needed to specifically test this scenario.

**Figure 6:**
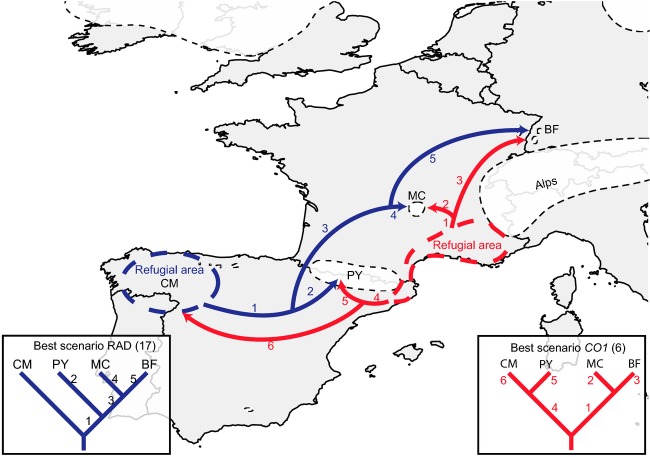
Recolonization routes of *Thremma gallicum* as inferred using ABC simulations for the RAD (blue) and mitochondrial *CO1* (red) datasets. Dashed areas indicate potential refugia for the respective scenarios. White space in dashed lines indicates the extent of glacial advances in Europe during the Last Glacial Maximum. Numbers on map refer to the number on branches in the phylogenetic trees. Drawings of glacial extent after Ehlers and Gibbard (2004).

The simulations of the population history performed with DIYABC separately for the RAD and *CO1* datasets support two competing scenarios (Table3, Fig.6). According to the RAD-based DIYABC results, *T. gallicum* first colonized the Pyrenees from the Cantabrian Mountains and then spread to both the Massif Central and the Black Forest later on. The mitochondrial data support a vicariance model with the most basal split between two groups of populations: the Pyrenees plus Cantabrian Mountains on the one hand and the Massif Central plus Black Forest on the other. Interestingly, the second best scenario identified by DIYABC for both marker sets corresponds to the best scenario for the complementary marker. This suggests that both scenarios can be explained by both datasets, albeit the support for the second scenario is considerably lower. It should be noted that both scenarios are quite similar and only differ by the placement of the root with otherwise compatible topologies. While the controversy cannot be fully resolved, it could be that the most recent common ancestor of all recent populations has diverged into an Iberian and a northern European clade that subsequently diverged further. For both models, the precise location of the ancestral populations cannot be identified, as the populations might be already extinct.

### How many individuals/loci are necessary?

A recurring question in population genetics is the minimum number of individuals and loci required to address a particular question of interest (e.g., Nei 1978; Koskinen et al. 2004; Willing et al. 2012). In a systematic meta-analysis for the standard mitochondrial *CO1* gene, Goodall-Copestake et al. (2012) assessed the minimum sampling per population needed to obtain reliable diversity estimates in single-marker *CO1* analyses, finding that at least 25 individuals are required. This condition was neither met in the present study nor in the majority of other studies on freshwater invertebrates. This lack of sampling can often be attributed to the difficulty of finding organisms, conservation concerns, or financial limitations. Aside from specimen number, it is also important to study patterns at several independent loci to avoid gene-specific biases (Ballard and Whitlock 2004). Analyses of genetic diversity (H and *π*) revealed contradicting results for the *CO1* dataset. While both nucleotide and haplotype diversity are lowest in the Massif Central, nucleotide diversity is highest in the Cantabrian Mountains, but haplotype diversity is highest in the Pyrenees. These results, however, could simply reflect the low number of analyzed specimens per stream and sampling sites for some regions. Only seven specimens from one sampling site could be analyzed for the Massif Central population, possibly explaining the low values for H and *π* found. But, sampling numbers aside, additional estimates of these important population genetic measures from other loci, especially those in the nuclear genome can greatly increase confidence in downstream results. RAD sequencing overcomes this challenge by sampling thousands of loci per individual and computing genetic diversity estimates averaged across all loci (observed heterozygosity), which is a far more reliable indicator of genetic diversity than mitochondrial haplotype and nucleotide diversity (Bazin et al. 2006; Galtier et al. 2009). The RAD dataset generated in this study revealed higher heterozygosity in putatively stable ancestral populations (i.e., Cantabrian Mountains, Massif Central) and lower values for those that are putatively younger or more impacted by disturbances (i.e., Black Forest and Pyrenees population). When testing for convergence of heterozygosity values across all 17 individuals included in the RAD dataset, it became obvious that a relatively low and feasible ODER easily sequenced number of loci is enough to make sound statements and get reliable results. Upon sampling only 250 loci for diversity estimates, the standard deviation had almost reached convergence (see Fig.4). In addition, we showed that individuals' patterns of diversity could be reliably differentiated with only 290 sampled loci for divergent populations like those from the Cantabrian Mountains and Black Forest (Fig.4B). Given the low number of sequenced specimens per population, it cannot be ruled out that outlier specimens with diversity patterns not representative for a population were sequenced. To reconstruct recent phylogeographic patterns from SNP data that show incomplete or only recently completed lineage sorting (as for the Massif Central and Black Forest populations), many more SNPs are needed to reach stable parameter estimates for our dataset with few individuals per site. For our study species, *T. gallicum*, the distance between the simulated and the optimal tree only converged when more than 3780 loci were included (Fig.5). Our data are consistent with the results of a recent simulation study that showed that even with sample sizes as low as *n* = 2, reliable differentiation estimates can be obtained when thousands of markers are genotyped (Willing et al. 2012). However, sequencing more individuals per population might change the observed pattern of genetic diversity, as outlier specimens could strongly influence the results.

## Conclusion and outlook

Very few studies have used both *CO1* and RAD data in non-model species (Emerson et al. 2010) for reconstructing a species' phylogeography. Here, we have shown that analysis of few individuals, but thousands of genome-wide SNPs in each individual, can improve the resolution and statistical robustness of phylogeographic inference. At the same time, RAD requires fewer specimens and sequencing can occur in a multiplexed, parallel fashion, making it both time and cost effective. In contrast to the large *CO1* dataset, the RAD data allowed for clearer identification of divergence and differentiation patterns in *T. gallicum* by utilizing thousands of nuclear markers and thus overcoming the inherent shortcomings of *CO1* data in cases of recurring gene flow following historic lineage separation. This study highlights the potential of RAD to reliably analyze genome-wide intraspecific genetic variation and demographic history even in non-model taxa. To the latter point, this combination of thousands of markers paired with ABC simulations makes it possible to test competing phylogeographic scenarios without the need to obtain an excessively large number of specimens or rely on one or a small number of loci each with their own independent evolutionary histories. For these reasons, RAD sequencing is a particularly interesting and promising tool for ecological and evolutionary studies for any taxonomic group, but especially for rare or endangered species with small population sizes.

## Data accessibility

*CO1* DNA sequences: GenBank accession numbers KP283027-KP283379. RAD data are available at the NCBI Short Read Archive (BioProject: PRJNA270569).

## Supporting Information

**Figure S1.***CO1* haplotype frequencies and distribution for the individual *Thremma gallicum* populations from the four distinct regions (Cantabrian Mountains, Pyrenees, Massif Central, and Black Forest) and Fct values between mountain ranges.

**Figure S2.** Isolation-by-distance analysis for the *CO1* data.

**Figure S3.** Phylogenetic tree with divergence dates (incl. 95% HPD intervals) calculated with BEAST v. 1.8.0 on the *CO1* data.

**Table S1.** Sampling sites, coordinates, number of specimens for *CO1* and RAD sequencing.

**Table S2.** Information on the specimens from different regions used for RAD analyses, number of Illumina reads, unique stacks, polymorphic loci and SNPs.

**Table S3.** Overview of divergence timings for different splits calculated using BEAST v. 1.8.0 on the *CO1* data using the molecular clock rate of 3.54% myr^-1^ as suggested by Papadopoulou et al.2010

## References

[b2] Arnold B, Corbett-Detig RB, Hartl D, Bomblies K (2013). RADseq underestimates diversity and introduces genealogical biases due to nonrandom haplotype sampling. Mol. Ecol.

[b3] Avise J (2000). Phylogeography: the history and formation of species.

[b4] Baird NA, Etter PD, Atwood TS (2008). Rapid SNP discovery and genetic mapping using sequenced RAD markers. PLoS One.

[b5] Bálint M, Barnard PC, Schmitt T, Ujvárosi L, Popescu O (2008). Differentiation and speciation in mountain streams: a case study in the caddisfly *Rhyacophila aquitanica* (Trichoptera). J. Zoolog. Syst. Evol. Res.

[b6] Bálint M, Domisch S, Engelhardt CHM, Haase P, Lehrian S, Sauer J (2011). Cryptic biodiversity loss linked to global climate change. Nat. Clim. Chang.

[b7] Ballard JW, Whitlock MC (2004). The incomplete natural history of mitochondria. Mol. Ecol.

[b8] Bazin E, Glémin S, Galtier N (2006). Population size does not influence mitochondrial genetic diversity in animals. Science.

[b9] Brito PH, Edwards SV (2009). Multilocus phylogeography and phylogenetics using sequence-based markers. Genetica.

[b10] Bunje PM (2005). Pan-European phylogeography of the aquatic snail *Theodoxus fluviatilis* (Gastropoda: Neritidae). Mol. Ecol.

[b11] Buoncristiani JF, Campy M (2004). Palaeogeography of the last two glacial episodes in the Massif Central, France. Dev. Quat. Sci.

[b12] Burton RS, Lee BN, Juan C, Oromi P, Hewitt GM (1994). Nuclear and mitochondrial gene genealogies and allozyme polymorphism across a major phylogeographic break in the copepod *Tigriopus californicus*. Proc. Natl Acad. Sci. USA.

[b13] Carstens B, Lemmon AR, Lemmon EM (2012). The promises and pitfalls of next-generation sequencing data in phylogeography. Syst. Biol.

[b14] Catchen JM, Amores A, Hohenlohe P, Cresko W, Postlethwait JH (2011). Stacks: building and genotyping loci de novo from short-read sequences. G3.

[b15] Catchen J, Bassham S, Wilson T (2013). The population structure and recent colonization history of Oregon threespine stickleback determined using restriction-site associated DNA-sequencing. Mol. Ecol.

[b16] Clement M, Posada D, Crandall KA (2000). TCS: a computer program to estimate gene genealogies. Mol. Ecol.

[b500] Cornuet JM, Pudlo P, Veyssier J, Dehne-Garcia A, Gautier M, Leblois R, Estoup A (2014). DIYABC v2. 0: a software to make approximate Bayesian computation inferences about population history using single nucleotide polymorphism. DNA sequence and microsatellite data. Bioinformatics.

[b17] Craft K, Pauls SU, Darrow K (2010). Population genetics of ecological communities with DNA barcodes: an example from New Guinea Lepidoptera. Proc. Natl Acad. Sci. USA.

[b18] Cutter AD (2013). Integrating phylogenetics, phylogeography and population genetics through genomes and evolutionary theory. Mol. Phylogenet. Evol.

[b19] Darriba D, Taboada GL, Doallo R, Posada D (2012). jModelTest 2: more models, new heuristics and parallel computing. Nat. Methods.

[b21] Drummond AJ, Ashton B, Buxton S (2011). http://www.geneious.com.

[b22] Drummond AJ, Suchard MA, Xie D, Rambaut A (2012). Bayesian phylogenetics with BEAUti and the BEAST 1.7. Mol. Biol. Evol.

[b23] Ehlers J, Gibbard PL (2004). Quaternary glaciations: extent and chronology part I: Europe.

[b24] Elbrecht V, Feld CK, Gies M (2014). Genetic diversity and dispersal potential of the stonefly *Dinocras cephalotes* in a central European low mountain range. Freshw. Sci.

[b25] Emerson BC, Oromí P, Hewitt GM (2000). Tracking colonization and diversification of insect lineages on islands: mitochondrial DNA phylogeography of *Tarphius canariensis* (Coleoptera: Colydiidae) on the Canary Islands. Proc. R. Soc. B.

[b26] Emerson KJ, Merz CR, Catchen JM (2010). Resolving postglacial phylogeography using high-throughput sequencing. Proc. Natl Acad. Sci. USA.

[b27] Excoffier L, Lischer HEL (2010). Arlequin suite ver 3.5: a new series of programs to perform population genetics analyses under Linux and Windows. Mol. Ecol. Resour.

[b28] Fijarczyk A, Nadachowska K, Hofman S (2011). Nuclear and mitochondrial phylogeography of the European fire bellied toads *Bombina bombina* and *Bombina variegata* supports their independent histories. Mol. Ecol.

[b29] Folmer O, Black M, Hoeh W, Lutz R, Vrijenhoek R (1994). DNA primers for amplification of mitochondrial *cytochrome c oxidase subunit I* from diverse metazoan invertebrates. Mol. Mar. Biol. Biotech.

[b30] Frohlich D, Torres-Jerez I, Bedford I, Markham P, Brown J (1999). A phylogeographical analysis of the *Bemisia tabaci* species complex based on mitochondrial DNA markers. Mol. Ecol.

[b31] Fulton TL, Norris RW, Graham RW, Semken HA, Shapiro B (2013). Ancient DNA supports southern survival of Richardson's collared lemming (*Dicrostonyx richardsoni*) during the last glacial maximum. Mol. Ecol.

[b32] Galtier N, Nabholz G, Glémin S, Hurst GDD (2009). Mitochondrial DNA as a marker of molecular diversity: a reappraisal. Mol. Ecol.

[b33] Goat1000 (2013). http://www.goat1000.com/svggraph.php.

[b34] Godinho R, Crespo E, Ferrand N (2008). The limits of mtDNA phylogeography: complex patterns of population history in a highly structured Iberian lizard are only revealed by the use of nuclear markers. Mol. Ecol.

[b35] Gomez A, Montero-Pau J, Lunt DHH, Serra M, Campillo S (2007). Persistent genetic signatures of colonization in *Brachionus manjavacas* rotifers in the Iberian Peninsula. Mol. Ecol.

[b36] González-Sampériz P, Leroy SA, Carrión JS (2010). Steppes, savannahs, forests and phytodiversity reservoirs during the Pleistocene in the Iberian Peninsula. Rev. Palaeobot. Palynol.

[b37] Goodall-Copestake WP, Tarling GA, Murphy EJ (2012). On the comparison of population-level estimates of haplotype and nucleotide diversity: a case study using the gene cox1 in animals. Heredity.

[b38] Hebert PDN, Stoeckle MY, Zemlak TS, Francis CM (2004). Identification of birds through DNA barcodes. PLoS Biol.

[b39] Hewitt GM (2000). The genetic legacy of the quaternary ice ages. Nature.

[b40] Ho SY (2007). Calibrating molecular estimates of substitution rates and divergence times in birds. J. Avian Biol.

[b41] Hohenlohe PA, Bassham S, Etter PD (2010). Population genomics of parallel adaptation in threespine stickleback using sequenced RAD tags. PLoS Genet.

[b42] Hohenlohe PA, Amish SJ, Catchen JM, Allendorf FW, Luikart G (2011). Next-generation RAD sequencing identifies thousands of SNPs for assessing hybridization between rainbow and westslope cutthroat trout. Mol. Ecol. Resour.

[b43] Hohenlohe PA, Bassham S, Currey M, Cresko WA (2012). Extensive linkage disequilibrium and parallel adaptive divergence across threespine stickleback genomes. Philos. Trans. R. Soc. Lond. B Biol. Sci.

[b44] Hughes PD, Woodward JC (2008). Timing of glaciation in the Mediterranean mountains during the last cold stage. J. Quat. Sci.

[b45] Hughes PD, Woodward JC, Gibbard PL (2006). Quaternary glacial history of the Mediterranean mountains. Prog. Phys. Geogr.

[b46] Huson DH, Bryant D (2006). Application of phylogenetic networks in evolutionary studies. Mol. Biol. Evol.

[b48] Jiménez-Sánchez M, Rodríguez-Rodríguez L, García-Ruiz JM, Domínguez-Cuesta MJ, Farias P, Valero-Garcés B (2013). A review of glacial geomorphology and chronology in northern Spain: timing and regional variability during the last glacial cycle. Geomorphology.

[b49] Jones JC, Fan S, Franchini P, Schartl M, Meyer A (2013). The evolutionary history of *Xiphophorus* fish and their sexually selected sword: a genome-wide approach using restriction site-associated DNA sequencing. Mol. Ecol.

[b50] Katoh K, Misawa K, Kuma K, Miyata T (2002). MAFFT: a novel method for rapid multiple sequence alignment based on fast Fourier transform. Nucleic Acids Res.

[b51] Kehl S (2005). *Thremma gallicum* McLachlan, 1880: Zur Biologie, Ökologie und Verbreitung einer faunistischen Besonderheit Deutschlands. Entomol. Heute.

[b52] Koskinen MT, Hirvonen H, Landry PAA, Primmer CR (2004). The benefits of increasing the number of microsatellites utilized in genetic population studies: an empirical perspective. Hereditas.

[b54] Lacourse T, Mathewes RW, Fedje DW (2005). Late-glacial vegetation dynamics of the Queen Charlotte Islands and adjacent continental shelf, British Columbia, Canada. Palaeogeogr. Palaeoclimatol. Palaeoecol.

[b55] Lehrian S, Pauls SU, Haase P (2009). Contrasting patterns of population structure in the montane caddisflies *Hydropsyche tenuis* and *Drusus discolor* in the Central European highlands. Freshw. Biol.

[b56] Lehrian S, Bálint M, Haase P, Pauls SU (2010). Genetic population structure of an autumn-emerging caddisfly with inherently low dispersal capacity and insights into its phylogeography. J. N. Am. Benthol. Soc.

[b57] Malicky H (1983). Chorological patterns and biome types of European Trichoptera and other freshwater insects. Arch. Hydrobiol.

[b58] Malicky H (2006). Mitteleuropäische (extra-mediterrane) Arealkerne des Dinodal am Beispiel von Köcherfliegen (Trichoptera). Beitr. Entomol.

[b59] Mardulyn P, Othmezouri N, Mikhailov YE, Pasteels JM (2011). Conflicting mitochondrial and nuclear phylogeographic signals and evolution of host-plant shifts in the boreo-montane leaf beetle *Chrysomela lapponica*. Mol. Phylogenet. Evol.

[b60] McCormack JE, Hird SM, Zellmer AJ, Carstens BC, Brumfield RT (2013). Applications of next-generation sequencing to phylogeography and phylogenetics. Mol. Phylogenet. Evol.

[b61] Nei M (1978). Estimation of average heterozygosity and genetic distance from a small number of individuals. Genetics.

[b62] Papadopoulou A, Anastasiou I, Vogler AP (2010). Revisiting the insect mitochondrial molecular clock: the mid-Aegean trench calibration. Mol. Biol. Evol.

[b63] Pauls SU, Lumbsch HT, Haase P (2006). Phylogeography of the montane caddisfly *Drusus discolor*: evidence for multiple refugia and periglacial survival. Mol. Ecol.

[b64] Penny D, Hendy M (1985). The use of tree comparison metrics. Syst. Zool.

[b65] Peterson BK, Weber JN, Kay EH, Fisher HS, Hoekstra HE (2012). Double digest RADseq: an inexpensive method for de novo SNP discovery and genotyping in model and non-model species. PLoS One.

[b66] Pujolar JM, Jacobsen M, Frydenberg J (2013). A resource of genome-wide single-nucleotide polymorphisms generated by RAD tag sequencing in the critically endangered European eel. Mol. Ecol. Resour.

[b501] Rambaut A (2007). http://tree.bio.ed.ac.uk/software/figtree.

[b67] Rambaut A, Suchard MA, Xie D, Drummond AJ (2013). http://beast.bio.ed.ac.uk/Tracer.

[b68] Reitzel A, Herrera S, Layden M, Martindale M, Shank T (2013). Going where traditional markers have not gone before: utility of and promise for RAD sequencing in marine invertebrate phylogeography and population genomics. Mol. Ecol.

[b69] Rousset F (2008). genepop'007: a complete reimplementation of the GENEPOP software for Windows and Linux. Mol. Ecol. Resour.

[b70] Salvi D, Harris DJ, Kaliontzopoulou A, Carretero MA, Pinho C (2013). Persistence across Pleistocene ice ages in Mediterranean and extra-Mediterranean refugia: phylogeographic insights from the common wall lizard. BMC Evol. Biol.

[b71] Schmitt T, Varga Z (2012). Extra-Mediterranean refugia: the rule and not the exception?. Front. Zool.

[b72] Schmitt T, Muster C, Habel JC, Assmann T, Schönswetter P (2010). Are disjunct alpine and arctic-alpine animal and plant species in the Western Palearctic really “Relics of a Cold Past”?. Relict species: phylogeography and conservation biology.

[b73] Senn H, Ogden R, Cezard T (2013). Reference-free SNP discovery for the Eurasian beaver from restriction site–associated DNA paired-end data. Mol. Ecol.

[b74] Serrano E, González-Trueba JJ, González-García M (2012). Mountain glaciation and paleoclimate reconstruction in the Picos de Europa (Iberian Peninsula, SW Europe). Quatern. Res.

[b75] Soltis DE, Morris AB, McLachlan JS, Manos PS, Soltis PS (2006). Comparative phylogeography of unglaciated eastern North America. Mol. Ecol.

[b76] Stewart JR, Lister AM, Barnes I, Dalén L (2010). Refugia revisited: individualistic responses of species in space and time. Proc. R. Soc. B.

[b77] Swofford D (2002). PAUP*. Phylogenetic analysis using Parsimony (*and Other Methods). version 4.

[b78] Taberlet P, Bouvet J (1994). Mitochondrial DNA polymorphism, phylogeography, and conservation genetics of the brown bear *Ursus arctos* in Europe. Proc. R. Soc. B.

[b80] Theissinger K, Bálint M, Feldheim KA, Haase P, Johannesen J, Laube I, Pauls SU (2013). Glacial survival and post-glacial recolonization of an arctic–alpine freshwater insect (*Arcynopteryx dichroa*, Plecoptera, Perlodidae) in Europe. J. Biogeogr.

[b81] White TA, Perkins SE, Heckel G, Searle JB (2013). Adaptive evolution during an ongoing range expansion: the invasive bank vole (*Myodes glareolus*) in Ireland. Mol. Ecol.

[b82] Willing E-V, Dreyer C, van Oosterhout C (2012). Estimates of genetic differentiation measures by FST do not necessarily require large sample sizes when using many SNP markers. PLoS One.

[b83] Wilson AC, Cann RL, Carr SM (1985). Mitochondrial DNA and two perspectives on evolutionary genetics. Biol. J. Linn. Soc.

