# Supplementary figures and images for: Assessing the phylogeographic history of the montane caddisfly Thremma gallicum using mitochondrial and restriction-site-associated DNA (RAD) markers
Source: Ecol Evol. 2015 Jan 13;5(3):648–62. doi: 10.1002/ece3.1366 (PMC4328769; doi:10.1002/ece3.1366)

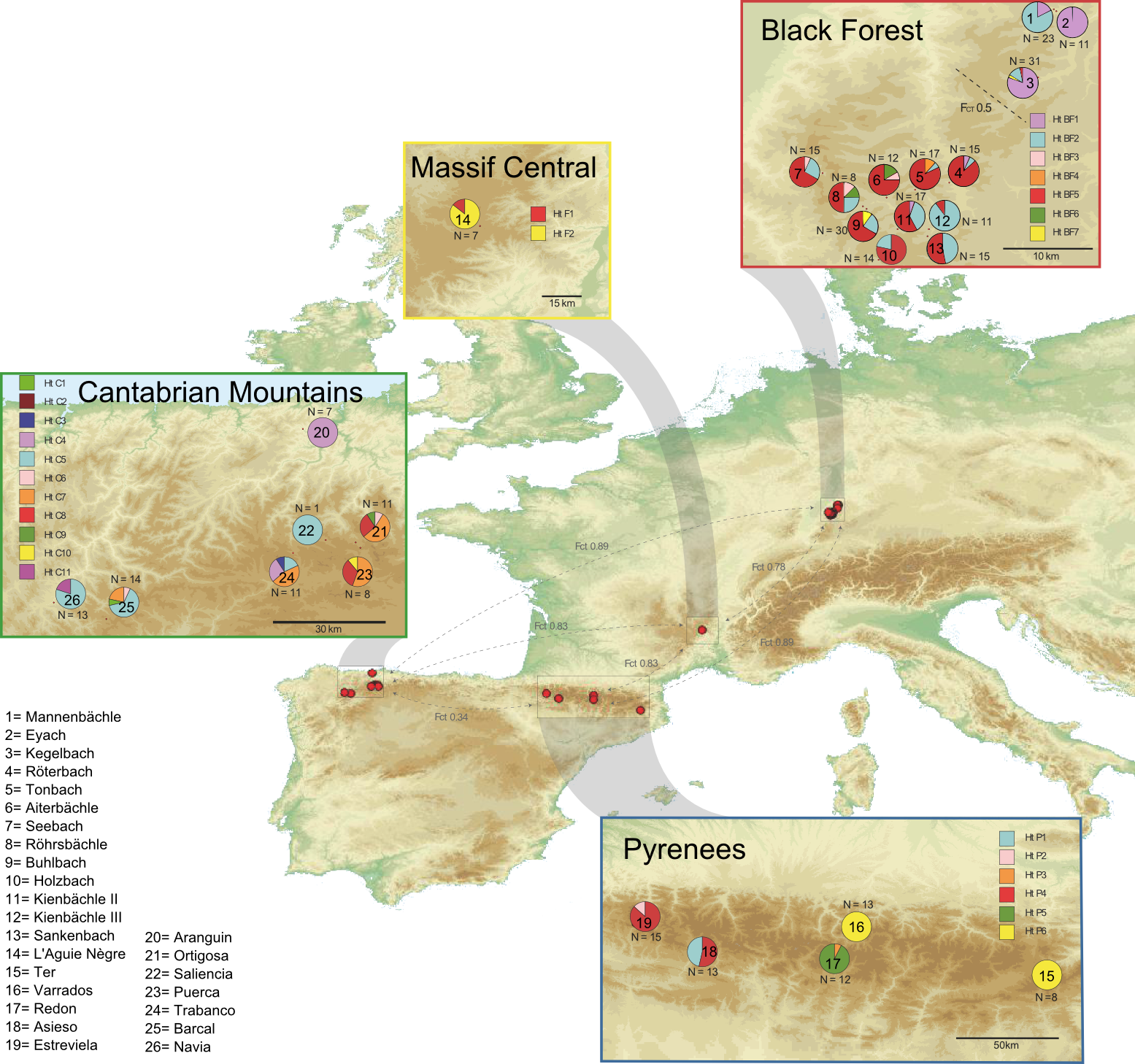

Supplement: Supplementary file 1 [file ece30005-0648-sd1.png]
