# Supplementary material for: Assessing the phylogeographic history of the montane caddisfly Thremma gallicum using mitochondrial and restriction-site-associated DNA (RAD) markers
Source: Ecol Evol. 2015 Jan 13;5(3):648–62. doi: 10.1002/ece3.1366 (PMC4328769; doi:10.1002/ece3.1366)
Supplement: Supplementary file 2 [file ece30005-0648-sd2.pdf]

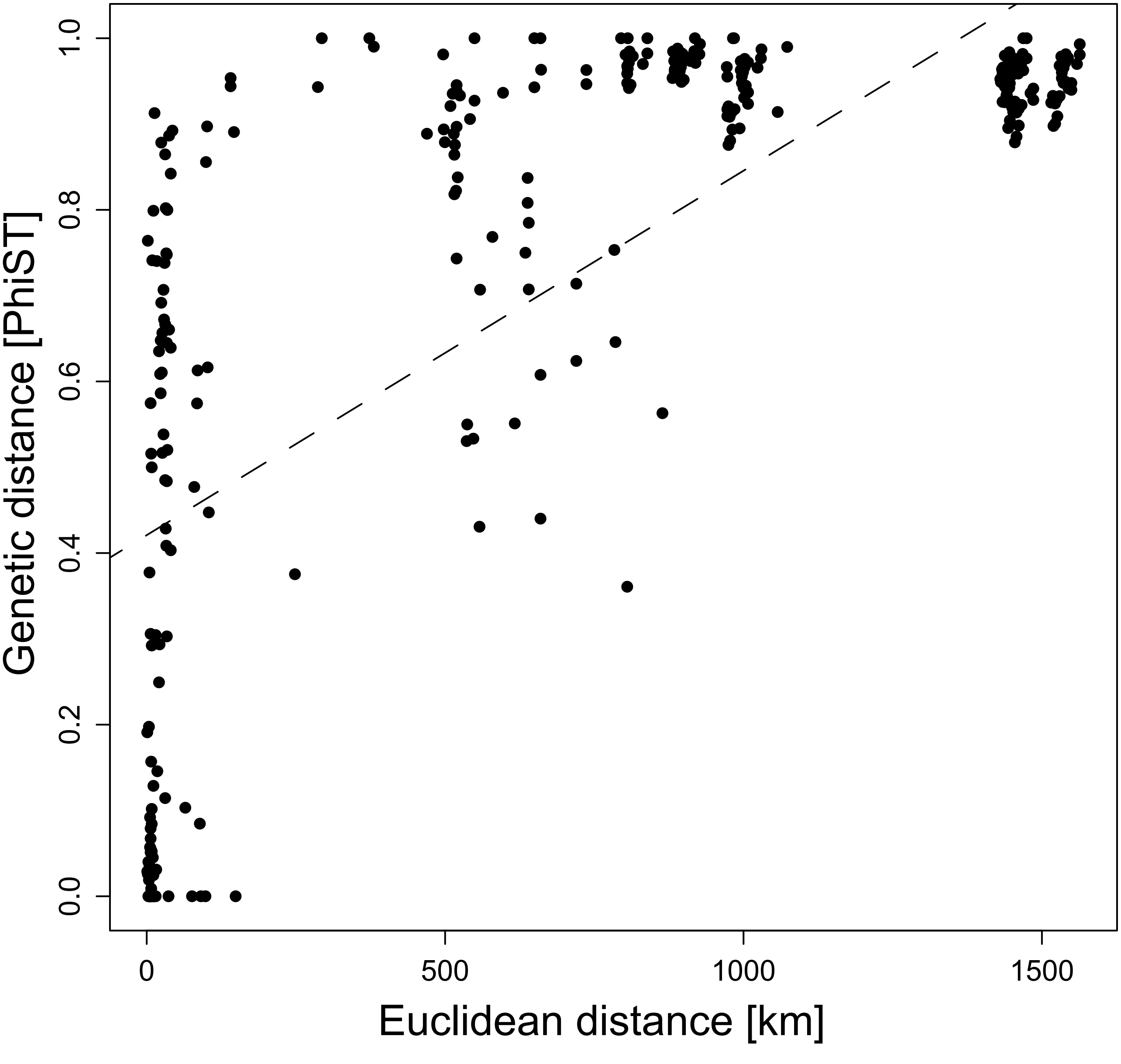

Figure S2: Isolation-by-distance analyses for the CO1 data. The underlying distance matrix (in km) of the sampling points was created using Quantum GIS v1.8 software (QGIS Development Team 2013) and then loaded into Arlequin. A Mantel test was applied to test for correlation between the Euclidean geographic distance matrix and pairwise population distance (PhiST) and significance was assessed by 10,000 random permutations. A strong positive correlation was observed ($p<0.001$).
