# Supplementary material for: Assessing the phylogeographic history of the montane caddisfly Thremma gallicum using mitochondrial and restriction-site-associated DNA (RAD) markers
Source: Ecol Evol. 2015 Jan 13;5(3):648–62. doi: 10.1002/ece3.1366 (PMC4328769; doi:10.1002/ece3.1366)
Supplement: Supplementary file 3 [file ece30005-0648-sd3.pdf]

Supporting information 6

Table S3: Overview over divergence timings for different splits calculated using BEAST v. 1.8.0 on the CO1 data using the molecular clock rate of 3.54% myr^{-1} as suggested by Papadopoulou *et al.* (2010). The population from the Cantabrian Mountains were paraphyletic (see Fig. S6 below).

Split	Time [myr BP]	95% HPD interval [myr BP]
MRCA	0.9247	1.5288 – 0.4254
Massif Central / Black Forest	0.2102	0.3802 – 0.0724
Iberian Peninsula 1	0.209	0.3712 – 0.0747
Iberian Peninsula 2	0.1498	0.2387 – 0.0475

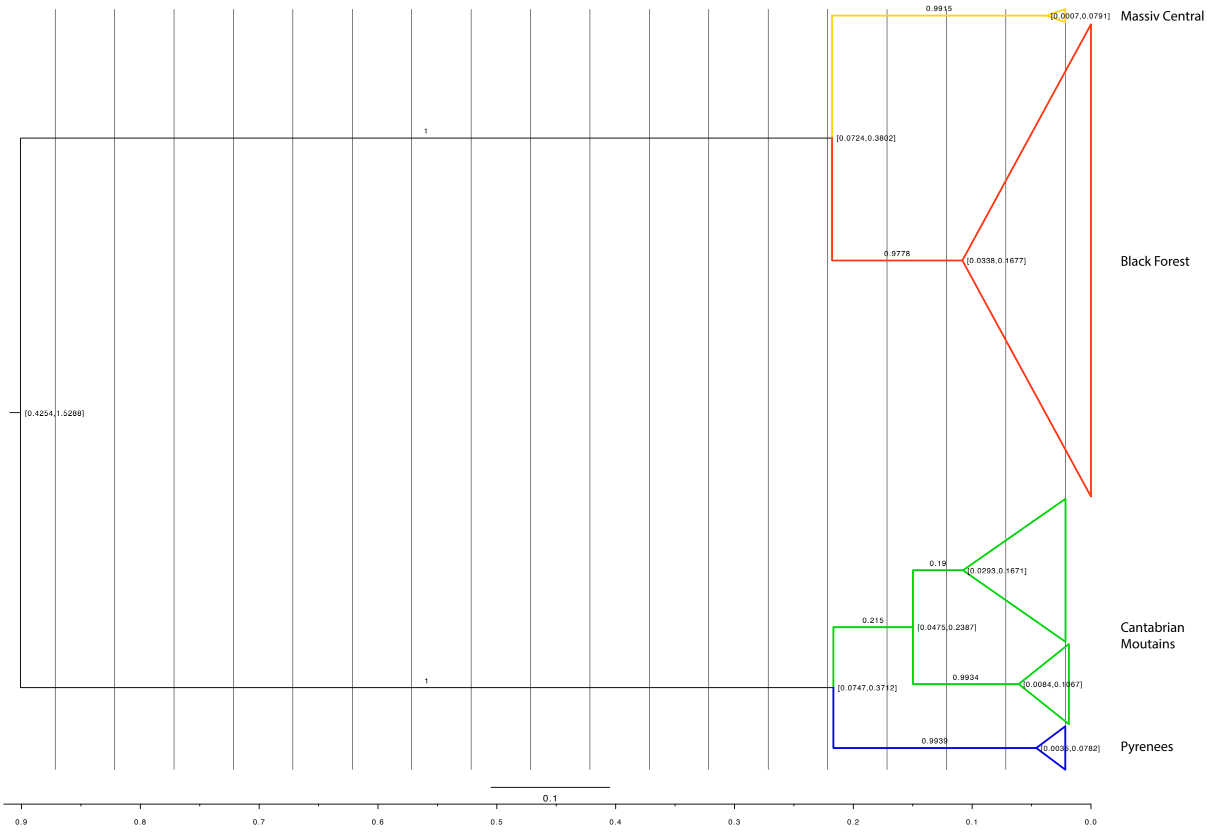

Figure S3: Phylogenetic tree with divergence dates (incl. 95% HPD intervals) calculated with BEAST v. 1.8.0 on the CO1 data. A molecular clock rate of 3.54% myr^{-1} was applied as suggested by Papadopoulou *et al.* (2010). Colours refer to the respective regions.
