# Supplementary material for: Assessing the phylogeographic history of the montane caddisfly Thremma gallicum using mitochondrial and restriction-site-associated DNA (RAD) markers
Source: Ecol Evol. 2015 Jan 13;5(3):648–62. doi: 10.1002/ece3.1366 (PMC4328769; doi:10.1002/ece3.1366)
Supplement: Supplementary file 4 [file ece30005-0648-sd4.pdf]

Table S1: Sampling sites, coordinates, number of specimens for *CO1* and RAD sequencing.

Sampling site	Coordinates	*CO1*: Number of specimens	RAD: Number of specimens
**Black Forest**			
Aiterbächle	48°32'19.87"N 8°18'1.66"E	12	
Buhlsbach	48°31'26.31"N 8°16'27.00"E	30	
Eyach	48°47'4.83"N 8°31'6.17"E	11	3
Holzbach	48°27'26.29"N 8°17'23.69"E	14	3
Kegelbach	48°41'44.05"N 8°29'52.78"E	31	
Kienbächle II	48°29'50.35"N 8°18'53.70"E	17	
Kienbächle III	48°29'51.20"N 8°21'19.29"E	11	
Mannenbächle	48°46'55.72"N 8°31'17.88"E	23	
Röhrsbach	48°31'38.34"N 8°16'9.46"E	8	4
Röterbach	48°33'29.84"N 8°22'55.14"E	15	
Sankenbach	48°29'17.57"N 8°21'14.21"E	15	
Seebach	48°34'14.19"N 8°13'0.78"E	15	
Tonbach	48°32'55.87"N 8°20'15.66"E	17	
**Massif Central**			
L'Aguie Nègre	44°49'50.87"N 4°11'50.96"E	7	2
**Pyrenees**			
Asieso	42°39'34.80"N 0°18'53.08"W	13	
Estreviela	42°49'49.30"N 0°42'44.20"W	15	1
Redon	42°38'12.80"N 0°46'43.30"E	12	1
Ter	42°20'16.92"N 2°18'39.52"E	8	
Varrados	42°46'27.00"N 0°47'4.30"E	13	
**Cantabrian Mountains**			
Aranguin	43°28'12.62"N 6°13'0.97"W	7	
Barcal	42°48'47.01"N 6°53'47.03"W	14	
Navia	42°52'10.12"N 7° 4'42.21"W	13	2
Ortigosa	43° 4'38.68"N 6° 2'2.15"W	11	
Puerca	43° 3'32.88"N 6° 1'3.83"W	8	1
Saliencia	43° 5'9.45"N 6° 8'15.89"W	1	
Trabanco	43° 2'27.36"N 6°15'1.13"W	11	
