# Supplementary material for: Assessing the phylogeographic history of the montane caddisfly Thremma gallicum using mitochondrial and restriction-site-associated DNA (RAD) markers
Source: Ecol Evol. 2015 Jan 13;5(3):648–62. doi: 10.1002/ece3.1366 (PMC4328769; doi:10.1002/ece3.1366)
Supplement: Supplementary file 5 [file ece30005-0648-sd5.pdf]

Table S2: Information on the specimens from different regions used for RAD analyses, number of Illumina reads, unique stacks, polymorphic loci and SNPs. BF=Black Forest, CM=Cantabrian Mountains, MC=Massif Central, PY=Pyrenees. For details see Table S1.

Sample	Species	Region	Barcode	Reads	Unique Stacks	Polymorphic Loci	SNPs found
Est2	*T. gallicum*	PY	GCCAAT	835701	60897	2934	4483
Ey4	*T. gallicum*	BF	ATCACG	1337427	141429	4380	6765
Ey8	*T. gallicum*	BF	TGACCA	1099580	120541	3635	5550
Ey9	*T. gallicum*	BF	AGTTCC	781254	80096	2878	4422
F3	*T. gallicum*	MC	GGCTAC	562982	49534	4266	6000
F8	*T. gallicum*	MC	CGTACG	1004398	92235	8032	10506
Ho2	*T. gallicum*	BF	GTTTCG	2990589	144491	5188	7620
Ho3	*T. gallicum*	BF	GTGAAA	1102120	102809	2730	4170
Ho4	*T. gallicum*	BF	GTGGCC	1532963	115092	3933	5786
Nav14	*T. gallicum*	CM	CGATGT	3819040	241947	35217	52098
Nav4	*T. gallicum*	CM	CAGATC	4706880	190410	26704	39563
Pu3	*T. gallicum*	CM	ACTTGA	1151892	118231	13725	19763
Red8	*T. gallicum*	PY	AGTCAA	2207080	160655	9460	13925
Roe18	*T. gallicum*	BF	ACAGTG	667452	85481	1776	2957
Roe3	*T. gallicum*	BF	GATCAG	304679	31121	809	1540
Roe5	*T. gallicum*	BF	GGTAGC	6044749	252309	11363	16171
Roe6	*T. gallicum*	BF	ACTGAT	926705	93497	2163	3489
TtPrad2	*T. tellae*		ATGAGC	1881539	83496	1718	2126
